# Evaluation of Aqueous Biphasic Electrophoresis System Based on Halide-Free Ionic Liquids for Direct Recovery of Keratinase

**DOI:** 10.3390/md19080463

**Published:** 2021-08-17

**Authors:** Phei Er Kee, Hip Seng Yim, Akihiko Kondo, John Chi-Wei Lan, Hui Suan Ng

**Affiliations:** 1 Faculty of Applied Sciences, UCSI University, UCSI Heights, Cheras, Kuala Lumpur 56000, Malaysia; 1001540680@ucsiuniversity.edu.my (P.E.K.); hsyim@ucsiuniversity.edu.my (H.S.Y.); 2 Biorefinery and Bioprocess Engineering Laboratory, Department of Chemical Engineering and Materials Science, Yuan Ze University, Chungli, Taoyuan 320, Taiwan; 3 Department of Chemical Science and Engineering, Graduate School of Engineering, Kobe University, 1-1 Rokkodai, Nada, Kobe 657-8501, Japan; akihiko.kondo@riken.jp; 4 Graduate School of Biotechnology and Bioengineering, Yuan Ze University, No. 135 Yuan-Tung Road, Chungli, Taoyuan 320, Taiwan

**Keywords:** aqueous biphasic electrophoresis system, halide-free, ionic liquids, electric fields, keratinase

## Abstract

Aqueous biphasic electrophoresis system (ABES) incorporates electric fields into the biphasic system to separate the target biomolecules from crude feedstock. Ionic liquid (IL) is regarded as an excellent candidate as the phase-forming components for ABES because of the great electrical conductivity, which can promote the electromigration of biomolecules in ABES, and thereby enhances the separation efficiency of the target biomolecules from crude feedstock. The application of electric fields to the conventional biphasic system expedites the phase settling time of the biphasic system, which eases the subsequent scaling-up steps and reduces the overall processing time of the recovery process. Alkyl sulphate-based IL is a green and economical halide-free surfactant when compared to the other halide-containing IL. The feasibility of halide-free IL-based ABES to recover *Kytococcus sedentarius* TWHK01 keratinase was studied. Optimum partition coefficient (K_e_ = 7.53 ± 0.35) and yield (Y_T_ = 80.36% ± 0.71) were recorded with IL-ABES comprised of 15.0% (*w*/*w*) [EMIM][ESO_4_], 20.0% (*w*/*w*) sodium carbonate and 15% (*w*/*w*) crude feedstock. Selectivity (S) of 5.75 ± 0.27 was obtained with the IL-ABES operated at operation time of 5 min with 10 V voltage supplied. Halide-free IL is proven to be a potential phase-forming component of IL-ABES for large-scale recovery of keratinase.

## 1. Introduction

Ionic liquids (ILs) are molten salts that appear in liquid state at room temperature with numerous unique features including non-volatility, non-flammability, low melting temperature, high thermal and electrochemical stabilities [1,2]. The IL with great solvation power has been widely used to recover and purify enzymes with enhanced catalytic activity, stability and selectivity [3,4]. Moreover, IL can be recycled after the extraction process through dialysis, absorption, membrane separation and crystallization and reused for subsequent extraction process, resulting in a lower processing cost required for the processes [5]. The IL can alter the polarity of the aqueous phases of the biphasic system because of the tunable cations and anions, thereby improving the extraction efficiency of the target biomolecules [4].

The IL containing halide anions such as chloride, bromide, iodide, tetrafluoroborate and hexafluoroborate has been commonly used to form biphasic system for biomolecules extraction [6]. However, the halide-containing IL demonstrates poor biodegradability and hazardous effects to the environment due to the release of toxic and corrosive by-products upon decomposition [7,8]. Therefore, alkyl sulphate-based IL is an emerging alternative, which is feasible for industrial applications because the synthesis process of halide-free IL can be performed under mild conditions in a one-pot reactor, at reasonable production cost with high yield [9,10]. The alkyl sulphate-based IL exhibits comparatively low viscosity, less toxicity and great biodegradability, resulting in the formation of environmentally benign biphasic system [10,11]. Moreover, alkyl sulphate-based IL is a promising electrolyte with excellent ionic conductivity [9], and is therefore ideal as an electrically conductive component for the biomolecules’ separation in the aqueous biphasic electrophoresis system (ABES).

ABES is an emerging technology which introduces the electric fields to the biphasic system for recovery of target biomolecules from crude feedstock [12]. Electric fields offer stability against convection in the biphasic system and allow a better electrokinetic mass transfer of charged proteins, and therefore improve the recovery of target biomolecules with respect to the yields and purity of the target biomolecules. For IL-based ABES, IL simultaneously acts as the electrolyte and extractant in the biphasic system, which facilitates the electrophoretic migration of the target biomolecules across the phase interface towards the preferred phase [13,14]. IL-based ABES is feasible at industrial level for the downstream processing of the target biomolecules with shortened processing time because of the enhanced phase separation rate of biphasic system with the imposition of electric fields [15].

Keratinase is an ecologically safe candidate in keratinous wastes management due to the capability in hydrolyzing the keratinous wastes into value-added bioproducts [16]. The production of keratinase via microbial fermentation only occurs in the supplementation of keratinous biomass as nutrient source, which can promote the recycling of keratinous wastes and minimize the environmental pollution issues [17]. Keratinase has been extensively used for diverse industrial applications ranging from animal feeds, organic fertilizers, cleaning agent formulation, leather and textile processing to pharmaceutical and cosmetic supplements [18]. The conventional keratinase purification approaches often require multiple stages of operation to attain desirable purity of keratinase with improved enzyme specificity and catalytic efficiency for commercial applications [19,20]. Therefore, there is a need to develop promising approaches that can effectively recover and purify keratinase with minimal processing time and cost.

Previous studies reported that IL-based ABES demonstrated better recovery yields and purity of keratinase when compared with the polymer/salt ABES [21]. In the present study, halide-free IL was used as the phase-forming component for the IL-based ABES with the aim to further reduce the cost and environmental hazards of the overall processing of the keratinase recovery. This is the first paper to date reporting on the feasibility of halide-free IL as the phase component of the ABES in the recovery of enzyme. The *Kytococcus sedentarius* TWHKC01 is a marine bacteria strain that demonstrates potential for the production of extracellular keratinase in the cultivation medium added with feather waste [15]. Hence, primary capture of the *K. sedentarius* TWHKC01 keratinase using biphasic system of alkyl sulphate-based IL was performed with the determination of several system parameters, including types of salt, phase composition and crude feedstock concentration. At a later stage, the feasibility of IL-based ABES on the recovery of *K. sedentarius* TWHKC01 keratinase was assessed by investigating the effects of the operation duration and operation voltage of the voltage supplier for the keratinase partition and recovery.

## 2. Results and Discussion

### 2.1. Binodal Curve of [EMIM][ESO_4_]/Salt Biphasic System

**Figure 1:**
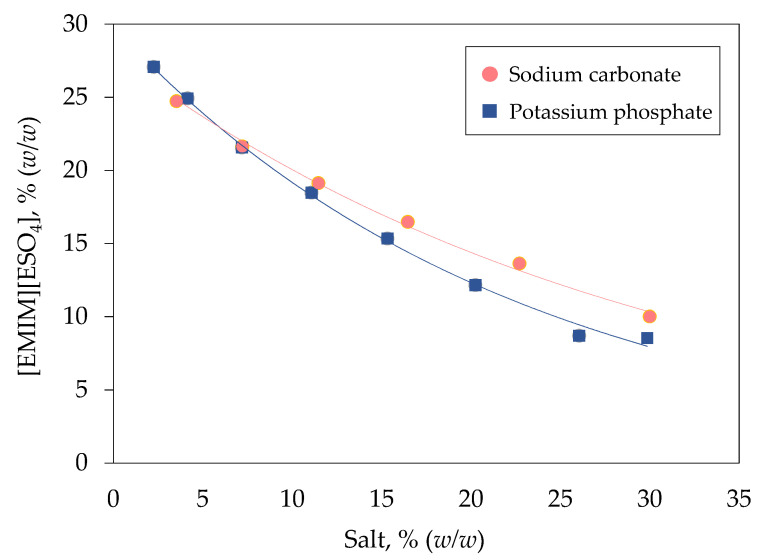
Binodal curve of [EMIM][ESO_4_]/salt biphasic system. The binodal curve for [EMIM][ESO_4_] was plotted against different concentration of sodium carbonate (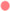) and potassium phosphate (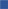).

Figure 1 illustrates the binodal curve of [EMIM][ESO_4_] with two different types of salt including carbonate and phosphate. Both carbonate and phosphate ions are kosmotropes, which possess a strong intermolecular interaction with water molecules, thereby reducing the amount of free water molecules for hydration of IL and inducing the phase separation [22]. The concentration of phase components needed for the formation of biphasic system generally relies upon the ions’ distribution of salt components [23]. A lower concentration of phase components is needed to form [EMIM][ESO_4_]/phosphate biphasic system because the binodal curve is nearer to the origin compared with the [EMIM][ESO_4_]/carbonate biphasic system. In IL-based biphasic system, the immiscibility of IL and salts is mainly caused by the salting-out effect of salt components, which could be corresponded to the Gibbs energy of hydration of ions (∆G_hyd_). Phosphate ions (∆G_hyd_ = −2765 kJ mol^−1^) exhibit greater salting-out ability than carbonate ions (∆G_hyd_ = −1315 kJ mol^−1^), leading to a stronger water-binding affinity to attract more water molecules toward them and form ion-hydration complexes [24]. Consequently, more water molecules are being excluded from the IL-rich phase, promoting the IL-based biphasic system formation [25].

### 2.2. Selection of Types of Salt for Keratinase Recovery

The effect of phosphate and carbonate salts on keratinase recovery in [EMIM][ESO_4_]-based biphasic system was examined, whereby the K_e_, Y_T_ and PF_T_ of keratinase are shown in Table 1. The concentration of IL and salt for the construction of biphasic system was fixed at 25% (*w*/*w*) and 15% (*w*/*w*), respectively, with the loading of 20% (*w*/*w*) crude feedstock into the biphasic system. Keratinase with isoelectric point of 2.7 acts as a negatively-charged protein in both the alkaline-based biphasic system with pH above its isoelectric point, thus migrating from salt-rich bottom phase to the IL-rich top phase to form electrostatic interaction with IL molecules [15]. Keratinase possessed a greater negative magnitude in the biphasic system of carbonate salt (pH 11) when compared with that of phosphate salt (pH 9), as increase in pH increases the surface charge negativity, resulting in a stronger electrostatic interaction among the positively-charged imidazolium cations with the negatively-charged keratinase in the IL-rich top phase [26].

The [EMIM][ESO_4_]-based biphasic system comprised of different types of salt that were constructed, and the recovery of keratinase were determined. The K_e_ and Y_T_ of keratinase were calculated based on Equations (1) and (6), respectively. The results are expressed as mean ± standard deviation of triplicate measurements.

In addition, different salting-out ability of salts affects the partition behavior of target enzyme in IL-based biphasic system in which the salt with stronger salting-out strength tends to promote the repellence of enzyme to the IL-rich top phase. Despite the greater salting-out strength of potassium phosphate as compared with sodium carbonate, the recovery efficiency of the biphasic system comprised of carbonate salt (K_e_ = 3.72 ± 0.24, Y_T_ = 80.60% ± 1.05 and PF_T_ = 1.21 ± 0.03) was better than that of phosphate salt (K_e_ = 1.37 ± 0.10, Y_T_ = 73.06% ± 1.34 and PF_T_ = 0.91 ± 0.03). Interphase precipitation was observed in the [EMIM][ESO_4_]/phosphate biphasic system, indicating the loss of proteins, which could be the possible reason for the low recovery of keratinase in IL-rich top phase. The carbonate salt was therefore selected as the salt component for keratinase recovery in [EMIM][ESO_4_]-based biphasic system.

### 2.3. Selection of Concentration of Phase Components for Keratinase Recovery

The effect of concentration of phase components on keratinase recovery in IL-based biphasic system was investigated by varying the concentration of [EMIM][ESO_4_] (15.0% (*w*/*w*) to 25.0% (*w*/*w*)) and carbonate (10.0% (*w*/*w*) to 25.0% (*w*/*w*)) (Table 2). The keratinase recovery in IL-rich top phase was decreased with the increment of IL concentration in biphasic system. In the biphasic system, comprised of 17.5% (*w*/*w*) carbonate, the K_e_ (from 4.94 ± 0.26 to 3.31 ± 0.33), Y_T_ (from 75.77% ± 0.99 to 74.45% ± 1.93) and PF_T_ (from 2.38 ± 0.06 to 1.41 ± 0.10) of keratinase were significantly reduced when [EMIM][ESO_4_] concentration was increased from 15.0% (*w*/*w*) to 25.0% (*w*/*w*). A similar trend was observed in biphasic system comprised of 20.0% (*w*/*w*) carbonate in which IL concentration of 20.0% (*w*/*w*) (K_e_ = 2.11 ± 0.06, Y_T_ = 65.32% ± 0.61, PF_T_ = 1.13 ± 0.05) and 25.0% (*w*/*w*) (K_e_ = 1.93 ± 0.23, Y_T_ = 62.85% ± 2.91, PF_T_ = 1.20 ± 0.06) resulted in lower recovery efficiency compared with that of 15.0% (*w*/*w*) (K_e_ = 5.29 ± 0.56, Y_T_ = 78.80% ± 1.50, PF_T_ = 2.38 ± 0.06). High IL concentration enhances the efficiency of biphasic system to recover biomolecules as a result of the formation of strong hydrophobic interaction among the target biomolecules and IL molecules [25,27]. Extreme high IL concentration was unfavorable for keratinase recovery, which could be due to the lowered stability and activity of keratinase enzyme [28].

The biphasic system, comprised of [EMIM][ESO_4_] and carbonate, was constructed at different concentrations, and the recovery of keratinase were determined. The K_e_, PF_T_ and Y_T_ of keratinase were calculated based on Equations (1), (5) and (6), respectively. The results are expressed as mean ± standard deviation of triplicate measurements.

Moreover, the keratinase recovery in the biphasic system comprised of 15.0% (*w/w*) [EMIM][ESO_4_] was improved with the increment of carbonate concentration from 17.5% (*w/w*) to 20.0% (*w/w*), followed by a decreasing trend when the carbonate concentration was further increased to 22.5% (*w/w*) and 25.0% (*w/w*). High salt concentration exhibits greater salting-out strength which drives more target biomolecules to migrate away from the salt-rich bottom phase to the IL-rich top phase [22]. The low efficiency of biphasic system with high salt concentration to recover keratinase is probably caused by the migration of excessive carbonate salt into the IL-rich top phase, thereby minimizing the amount of free water molecules available in IL-rich top phase to interact with keratinase enzyme, leading to the transfer of keratinase to the salt-rich bottom phase [29]. Hence, the highest recovery efficiency was achieved in the biphasic system constituting 15.0% (*w/w*) [EMIM][ESO_4_] and 20.0% (*w/w*) carbonate with K_e_ of 5.29 ± 0.56, Y_T_ of 78.80% ± 1.50 and PF_T_ of 2.38 ± 0.06 recorded.

### 2.4. Selection of the Amount of Crude Feedstock Load for Keratinase Recovery

**Figure 2:**
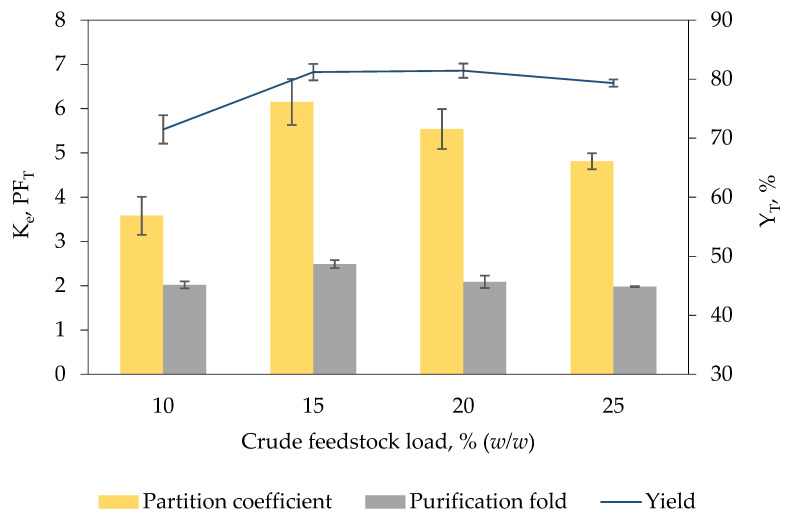
Effect of the amount of crude feedstock load on K_e_, PF_T_ and Y_T_ of keratinase. The amount of crude feedstock loaded into the biphasic system for optimal keratinase recovery was determined by adding different amounts of *K. sedentarius* crude feedstock (10% (*w*/*w*) to 25% (*w*/*w*)) into the [EMIM][ESO_4_]/carbonate biphasic system. The K_e_, PF_T_ and Y_T_ of keratinase were calculated based on Equations (1), (5) and (6), respectively. The results are expressed as mean of triplicate measurements, and error bar represents ± standard deviation.

Figure 2 shows the efficiency of [EMIM][ESO_4_]/carbonate biphasic system loaded with different concentration of crude *K. sedentarius* TWHKC01 feedstock varying from 10% (*w*/*w*) to 25% (*w*/*w*) on keratinase recovery. Increase in crude feedstock concentration increases the content of target enzymes added into the biphasic system, thereby enhancing the recovery of target enzymes in IL-rich top phase [23]. The finding was in accordance with the aforementioned statement whereby an increasing trend was demonstrated as the concentration of crude feedstock increased from 10% (*w/w*) (K_e_ = 3.58 ± 0.43, Y_T_ = 71.48% ± 2.41, PF_T_ = 2.02 ± 0.08) to 15% (*w*/*w*) (K_e_ = 6.15 ± 0.52, Y_T_ = 81.19% ± 1.39, PF_T_ = 2.49 ± 0.09).

On the contrary, further increment of concentration of crude feedstock to 20% (*w*/*w*) (K_e_ = 5.54 ± 0.45, Y_T_ = 81.43% ± 1.21, PF_T_ = 2.09 ± 0.14) and 25% (*w*/*w*) (K_e_ = 4.81 ± 0.18, Y_T_ = 79.33% ± 0.62, PF_T_ = 1.98 ± 0.01) reduced the keratinase recovery. High crude feedstock concentration often corresponds to high impurity content which gives rise to the alteration of phase composition and electrostatic potential of biphasic system [22]. The impurities might transfer to the IL-rich top phase forming electrostatic interaction with the IL molecules, consequently minimizing the free space available in the IL-rich top phase to accommodate keratinase molecules [21]. The biphasic system added with 15% (*w*/*w*) crude feedstock demonstrated the highest recovery efficiency, indicating that the maximum solubility of keratinase in IL-rich top phase of the developed biphasic system has been achieved.

### 2.5. Selection of Operation Time for Keratinase Recovery

**Figure 3:**
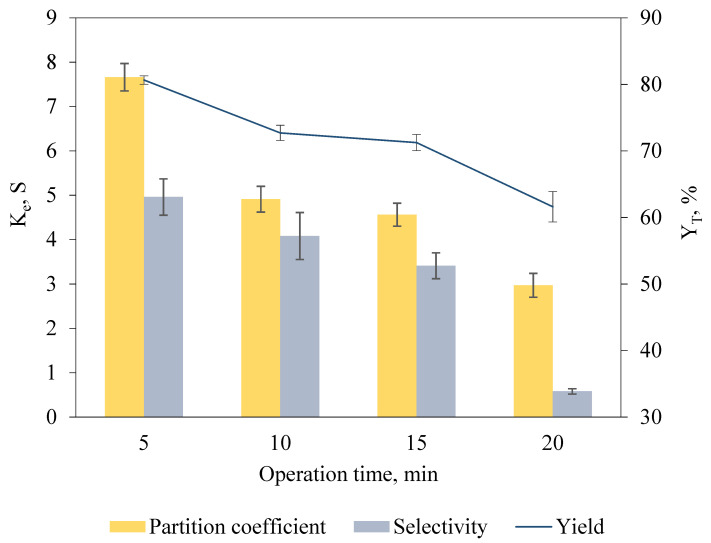
Effect of operation time of ABES on K_e_, S and Y_T_ of keratinase. Electric fields were incorporated into the [EMIM][ESO_4_]/carbonate biphasic system at different durations (5 min to 20 min) for keratinase recovery. The K_e_, S and Y_T_ of keratinase were calculated based on Equations (1), (3) and (6), respectively. The results are expressed as mean of triplicate measurements, and error bar represents ± standard deviation.

To identify the operation time for optimal keratinase recovery in the biphasic system incorporated with anode in salt-rich bottom phase and cathode in IL-rich top phase, operation durations differing from 5 min to 20 min were studied (Figure 3). A longer operation time offers sufficient time for complete phase separation and lengthens the duration allowing for the charged target biomolecules to migrate across the phase interface to the IL-rich top phase [15]. However, a longer operation time was observed to possess an adverse effect on the recovery efficiency whereby the K_e_, Y_T_ and S recorded were notably decreased. The operation time of 5 min resulted in maximum K_e_ of 7.66 ± 0.31, Y_T_ of 80.65% ± 0.64 and S of 4.96 ± 0.41, indicating that short duration was sufficient for complete phase separation and promoted the mass transfer of keratinase toward the IL-rich top phase.

Lower K_e_, Y_T_ and S of keratinase were recorded when the operation time was further increased to 10 min (K_e_ = 4.91 ± 0.29, Y_T_ = 72.70% ± 1.15, S = 4.08 ± 0.53), 15 min (K_e_ = 4.56 ± 0.26, Y_T_ = 71.25% ± 1.21, S = 3.41 ± 0.29) and 20 min (K_e_ = 2.97 ± 0.22, Y_T_ = 61.60% ± 2.29, S = 0.58 ± 0.06). A prolonged operation time causes the accumulation of biomolecules at the phase interface, leading to the inverse concentration diffusion, and limits the transfer of keratinase to IL-rich top phase because of the high interfacial tension of the biphasic system [30]. Additionally, the low S of keratinase recorded as the operation time increases could be caused by the migration of unwanted protein contaminants to the IL-rich top phase, suggesting that long operation time was unfavorable for keratinase recovery. 

### 2.6. Selection of Operation Voltage for Keratinase Recovery

**Figure 4:**
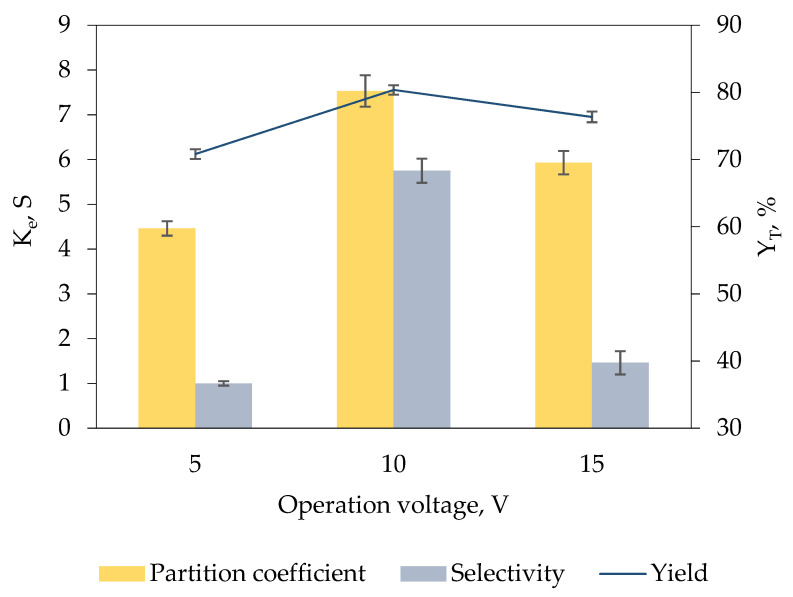
Effect of operation voltage of ABES on K_e_, S and Y_T_ of keratinase. Electric fields of different voltage (5 V to 15 V) were incorporated into the [EMIM][ESO_4_]/carbonate biphasic system for 5 min for keratinase recovery. The K_e_, PF_T_ and Y_T_ of keratinase were calculated based on Equations (1), (5) and (6), respectively. The results are expressed as mean of triplicate measurements, and error bar represents ± standard deviation.

Figure 4 demonstrates the effect of operation voltage ranging from 5 V to 15 V on the recovery of keratinase. High voltage exerts a greater force on the charged biomolecules, allowing the biomolecules to overcome the impedance of interphase and more readily to be migrated to one of the specific phases in the biphasic system [30,31]. The efficiency of the biphasic system for keratinase recovery was significantly enhanced with the increment of operation voltage from 5 V (K_e_ = 4.46 ± 0.16, Y_T_ = 70.81% ± 0.73, S = 1.00 ± 0.05) to 10 V (K_e_ = 7.53 ± 0.35, Y_T_ = 80.36% ± 0.71, S = 5.75 ± 0.27). This proves that the electromigration of keratinase to the IL-rich top phase is facilitated by the stronger electric field strength in the biphasic system; hence, a better recovery performance was attained. 

The operation voltage of 10 V was determined as the optimal voltage for keratinase recovery in the developed biphasic system because further increase in operation voltage to 15 V exhibited a poor recovery efficiency with comparatively low K_e_ of 5.93 ± 0.26, Y_T_ of 76.34% ± 0.79 and S of 1.46 ± 0.26 obtained. High electric fields cause the formation of perturbations at phase interface, which leads to the instability of phase interface, thereby influencing the mass transfer of enzymes in the biphasic system [32,33].

### 2.7. Determination of Purity of Keratinase Recovered from the Halide-Free IL-Based ABES

**Figure 5:**
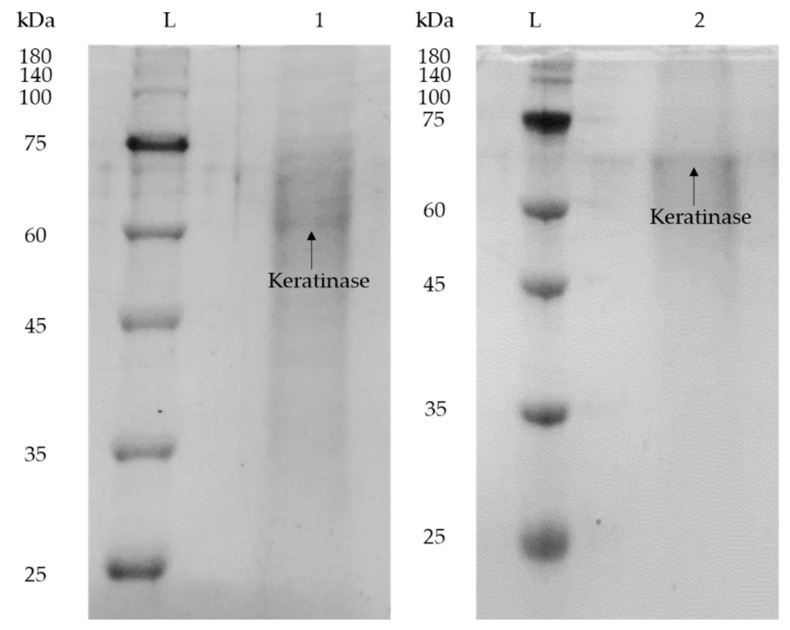
SDS-PAGE profile of crude and purified enzyme. Lane L: pre-stained protein ladder; Lane 1: crude *K. sedentarius* TWHKC01 keratinase; Lane 2: purified *K. sedentarius* TWHKC01 keratinase in IL-rich top phase sample.

The purity of keratinase recovered from the top phase of the system was assessed with SDS-PAGE analysis and with comparison to the crude *K. sedentarius* TWHCK01 keratinase, as shown in Figure 5. Lane L indicates pre-stained protein ladder, Lane 1 indicates crude keratinase and Lane 2 indicates purified keratinase. The presence of keratinase and other protein contaminants in *K. sedentarius* TWHKC01 crude feedstock was confirmed through the observation of multiple distinct bands in Lane 1. On the other hand, a clear band was observed in Lane 2, indicating that the keratinase was successfully purified in the IL-rich top phase with the removal of other protein contaminants to the salt-rich bottom phase. This is in agreement with the molecular weight of *K. sedentarius* TWHKC01 stated in the published literature, which was 60 kDa [15]. The slight discrepancy in the position of band shown in Lane 2 is probably due to the minimal binding of SDS to protein samples as a consequence of the high ionic strength of IL in the top phase sample. Additionally, the presence of IL in the top phase sample, which formed an electrostatic interaction with proteins, caused an increase in the proteins’ size, resulting in a protein band shift in the top phase sample [26]. Therefore, the proposed IL-based ABES is proven as an effective purification technology for the keratinase production.

## 3. Materials and Methods

### 3.1. Materials

The 1-ethyl-3-methylimidazolium ethyl sulphate, [EMIM][ESO_4_] (≥95.0% purity) was acquired from Sigma-Aldrich (St. Louis, MO, USA). Dipotassium hydrogen phosphate, potassium dihydrogen phosphate and sodium carbonate were supplied by Merck (Darmstadt, Germany). All of the analytical grade (AR) chemicals were used in the present study.

### 3.2. Microbial Fermentation of Kytococcus sedentarius TWHKC01 for Keratinase Production

The cultivation medium was prepared based on the formulation as detailed in previous published work [15]. The keratinase fermentation was performed by inoculating 10% (*v/v*) bacterial inoculum into the Erlenmeyer flask containing sterilized cultivation medium with a total working volume of 100 mL. The crude feedstock was harvested after the incubation of inoculated medium at 37 °C for 72 h with 200 rpm of agitation speed, followed by centrifugation at cold temperature (4 °C) for 10 min at 8944× *g*.

### 3.3. Construction of Binodal Curve

Turbidimetric titration method was employed to construct the binodal curve of [EMIM][ESO_4_] with two types of salt (i.e., sodium carbonate and potassium phosphate) [23]. The biphasic system of 2 g with different phase composition was prepared by mixing known amount of IL and salts. A cloudy mixture was formed, implying the formation of two phases. Then, the mixture was added with distilled water dropwise until the mixture turned clear, implying the formation of one phase. The weight of distilled water added for the disappearance of turbidity was recorded. The final composition of phase components were calculated and the binodal curve was constructed. 

### 3.4. Partition Experiment

The 20 g IL-based biphasic system was constructed by mixing the proper weight of [EMIM][ESO_4_], salts, crude feedstock and distilled water in a 50 mL falcon tube. Electric fields were incorporated into the biphasic system by placing anode and cathode in the salt-rich bottom phase and IL-rich top phase of biphasic system, respectively. The operation voltage was set at 10 V for 5 min, unless stated otherwise. The top and bottom phases’ volume were determined, followed by the collection of phase samples for evaluation of total protein content and keratinolytic enzyme activity. The optimum condition of biphasic system for keratinase recovery was determined by investigating the effect of types of salt component, concentration of phase components, crude feedstock loading, operation time and operation voltage [15].

### 3.5. Keratinolytic Activity Assay and Bicinchoninic Acid Assay

The keratinase enzyme activity and total protein content in the phase sample were assessed using keratinolytic activity assay and bicinchoninic acid (BCA) assay, respectively, by referring to the procedure mentioned in previous studies [15,21]. Briefly, the keratinolytic activity assay was performed by incubating the mixture of enzyme-containing sample and 0.5% (*w/v*) keratin substrate solution in a water bath (40 °C, 30 min). The mixture was incubated again (4 °C, 10 min) after the termination of reaction with 10% (*w/v*) trichloroacetic acid (TCA), followed by the centrifugation of reaction mixture and the measurement of absorbance of supernatant (280 nm) [34]. BCA assay was carried out using Pierce^TM^ BCA Protein Assay Kit (Themo Fisher Scientific, Waltham, MA, USA) in which the mixture of protein-containing sample and BCA working reagent was incubated (37 °C, 30 min), continued by the measurement of the absorbance (562 nm).

### 3.6. Determination of Recovery Yield, Partition Coefficient, Selectivity and Purification Fold

Partition coefficient of keratinase enzyme (K_e_) was determined based on Equation (1) [35]:

(1) $$K_{e}=\frac{e_{T}}{e_{B}}$$

where $e_{T}$ represents the keratinase activity in the top phase and $e_{B}$ represents the keratinase activity in the bottom phase.

Partition coefficient of total protein (K_p_) was determined based on Equation (2) [35]:

(2) $$K_{p}=\frac{p_{T}}{p_{B}}$$

where $p_{T}$ represents the total protein content in the top phase and $p_{B}$ represents the total protein content in the bottom phase, respectively.

Selectivity (S) was evaluated using Equation (3) [21]:

(3) $$S=\frac{K_{e}}{K_{p}}$$

Specific enzyme activity (SA) of keratinase was calculated using Equation (4) [22]:

(4) $$\mathrm{SA}=\frac{\mathrm{Keratinase}\;\mathrm{enzyme}\;\mathrm{activity}\;\left(U\right)}{\mathrm{Total}\;\mathrm{protein}\;\mathrm{content}\;\left(\mathrm{mg}\right)}$$

Purification fold (PF_T_) of keratinase was determined according to Equation (5) [22]:

(5) $${\mathrm{PF}}_{T}=\frac{\mathrm{SA}\;\mathrm{in}\;\mathrm{top}\;\mathrm{phase}\;\left({\mathrm{SA}}_{T}\right)}{\mathrm{SA}\;\mathrm{in}\;\mathrm{crude}\;\mathrm{feedstock}\;\left({\mathrm{SA}}_{C}\right)}$$

The percentage yield of keratinase (Y_T_) in IL-rich top phase was determined based on Equation (6) [22]:

(6) $$Y_{T}=\frac{100}{1+\left(\frac{1}{V_{r}+K_{e}}\right)}$$

where $V_{r}$ denotes the volume ratio of biphasic system. 

### 3.7. Sodium Dodecyl Sulphate-Polyacrylamide Gel Electrophoresis (SDS-PAGE) Analysis

Trichloroacetic acid (TCA) precipitation method was employed to precipitate and concentrate the protein samples prior to the SDS-PAGE analysis [21]. SDS-PAGE analysis was carried out by referring to the procedure mentioned in a previous study using ATTO electrophoresis unit (Tokyo, Japan) [15].

## 4. Conclusions

Halide-free IL was practicable for IL-based ABES to recover *K. sedentarius* TWHKC01 keratinase from crude feedstock. IL-based ABES constituted of 15.0% (*w*/*w*) [EMIM][ESO_4_], 20.0% (*w*/*w*) carbonate and 15% (*w*/*w*) crude feedstock was determined as the optimum condition for keratinase recovery with K_e_ of 7.53 ± 0.35 recorded. Keratinase was purified in the IL-rich top phase with S of 5.75 ± 0.27 and Y_T_ of 80.36% ± 0.71 when the operation voltage was set at 10 V for a duration of 5 min. IL-based ABES could be employed as a promising alternative for the single-step recovery of microbial keratinase at industrial level with minimal processing cost and time. Despite having a lower recovery efficiency when compared with the IL-based ABES with 1-butyl-3-methylimidazolium tetrafluoroborate applied in the previous study (Y_T_ of 95.82% and S of 10.87), alkyl sulphate-based IL is recommended as an ideal component for the construction of green and cost-effective IL-based ABES because of the halide-free properties and can be obtained at a lower cost. The IL-based ABES constructed in the present study requires a lower amount of IL and salts when compared with the IL-based ABES constructed in the previous study. The application of lesser phase-forming reagents and the adoption of cheaper and more environmentally friendly measures result in a more sustainable and cost-effective approach for the practical implementation of large-scale recovery of keratinase.

## Figures and Tables

**Table 1:** Effect of types of salt on K_e_, Y_T_ and PF_T_ of keratinase.

Phase Component		K_e_	Y_T_, %	PF_T_
IL	Salt			
[EMIM][ESO_4_]	Carbonate	3.72 ± 0.24	80.60 ± 1.05	1.21 ± 0.03
[EMIM][ESO_4_]	Phosphate	1.37 ± 0.10	73.06 ± 1.34	0.91 ± 0.03

**Table 2:** Effect of concentration of phase components on K_e_, Y_T_ and PF_T_ of keratinase.

Concentration, % (*w*/*w*)		K_e_	Y_T_, %	PF_T_
[EMIM][ESO_4_]	Carbonate			
15.0	17.5	4.94 ± 0.26	75.77 ± 0.99	2.07 ± 0.04
	20.0	5.29 ± 0.56	78.80 ± 1.50	2.38 ± 0.06
	22.5	2.63 ± 0.11	59.71 ± 0.99	2.30 ± 0.09
	25.0	2.02 ± 0.14	53.17 ± 1.70	1.98 ± 0.07
20.0	15.0	3.29 ± 0.31	74.38 ± 1.90	1.45 ± 0.11
	17.5	4.48 ± 0.18	78.12 ± 0.68	1.47 ± 0.05
	20.0	2.11 ± 0.06	65.32 ± 0.61	1.13 ± 0.05
	22.5	1.44 ± 0.04	56.09 ± 0.75	1.00 ± 0.05
25.0	10.0	1.78 ± 0.10	75.81 ± 1.00	1.28 ± 0.03
	12.5	2.87 ± 0.20	76.12 ± 1.24	1.57 ± 0.06
	15.0	4.44 ± 0.23	81.54 ± 0.76	1.46 ± 0.05
	17.5	3.31 ± 0.33	74.45 ± 1.93	1.41 ± 0.10
	20.0	1.93 ± 0.23	62.85 ± 2.91	1.20 ± 0.06
